# Role of Platelet Indices as a Potential Marker for Malaria Severity

**DOI:** 10.1155/2021/5531091

**Published:** 2021-03-16

**Authors:** Biruk Bayleyegn, Fikir Asrie, Aregawi Yalew, Berhanu Woldu

**Affiliations:** Department of Hematology and Immunohematology, College of Medicine and Health Science, University of Gondar, P.O. Box 196, Gondar, Ethiopia

## Abstract

**Purpose:**

Platelet parameter alteration such as platelet count and platelet indices are more common than in other blood cell lines due to diverse causative pathophysiological mechanisms in severe malaria infection. In malaria patients, no more studies evaluated platelet indices in relation to disease severity and prognosis. Therefore, this review assessed the current scientific knowledge on the potential role of platelet indices for the diagnostic marker of severe malaria infection.

**Results:**

Hence, after reviewing recent literatures, elevation of mean platelet volume and platelet distribution width in addition to decreased plateletcrit and platelet counts is the known potential risk factor associated with warning signs of severe malaria. Thus, thrombocytopenia < 150 × 10^9^/L, MPV ≥ 9.05 fL, and PDW ≥ 14.550% as well as significantly higher P-LCR and decrease in PCT are shown significant sensitivity and specificity as they are used as diagnostic and prognostic values in severe malaria infection.

**Conclusion:**

Platelet indices are useful predictors of malaria severity. Immature platelet fraction (IPF%) is raised in the case of severe malaria, and it was significantly more useful than MPV. Advanced research will further investigate the platelet index abnormality associated with specific age and gender among specific malaria species.

## 1. Introduction

Malaria is a blood parasitic disease caused by protozoans of five plasmodium species and transmitted by infected female anopheles mosquito. The different plasmodia are not uniformly distributed throughout the world. *Plasmodium vivax* (PV) is more predominant in Asia and *Plasmodium falciparum* (PF) in Africa [1]. Severe malaria by definition is associated with a high mortality and reported as the fifth cause of death from other infectious diseases in the globe, and the second leading cause of death in the sub-Saharan region including Ethiopia. It is mainly caused by PF, although *Plasmodium knowlesi* and PV may also cause severe illness and result in death. An extremely high number of severe malaria illnesses and deaths occur especially in children under 5 years, elderly, and pregnant women before visiting a health service [2–4]. Severe malaria infection is distinguished from uncomplicated malaria by a set of diagnostic criteria, including clinical and laboratory indicators [5, 6].

Hematological abnormalities are the most common complications during severe malaria infections and play a major role in the fatality. Level of malaria endemicity, background hemoglobinopathy, demographic factors, nutritional status, and malaria immunity are factors for hematological alterations associated with malaria infection [7, 8]. Thrombocytopenia, which is characterized by decreased platelet levels (<150 × 103/*μ*L), is a common hematological alteration during malaria infections. Thrombocytopenia seems to occur primarily by peripheral destruction, bone marrow alterations, excessive removal of platelets by the spleen, platelet consumption by the process of disseminated intravascular coagulopathy [9], and pseudothrombocytopenia due to clumping of PF-infected erythrocytes [10, 11]. In addition, the underlying mechanisms of thrombocytopenia in malaria are destruction of platelets by IgG antibodies, release of adenosine diphosphate (ADP) by hemolyzed parasitized RBCs, dysmegakaryopoiesis, direct lytic effect of the parasite on the platelets, platelet phagocytosis, platelet adhesion to erythrocytes, and oxidative stress [12–14]. These possible pathophysiological mechanisms that lead to platelet homeostasis are more predominant than other blood cell abnormality. In malaria-infected patients, thrombocytopenia is observed to improve with disease resolution and a normal platelet number is usually detected 7 days after initiation of specific antimalaria drugs [15]. Not only the reduction of platelet counts but also the function of platelets is compromised in malaria-infected patients especially in complicated cases. According to different medical literature reports, this is generally evidenced by changes in the volume and other features of platelet cells [9, 16]. Thrombocytopenia and abnormality of the platelet indices are the common features of complicated malaria infections. Furthermore, platelet activation alters the platelet parameters including mean platelet volume (MPV), platelet distribution width (PDW), platelet large cell ratio (P-LCR), and plateletcrit (PCT) which are reliable indicators of platelet biomass in malaria-infected patients. All of these indices are considered as markers of platelet activation and significantly altered in patients with malaria infection [17, 18].

The gold standard method for malaria diagnosis is microscopic examination by identifying and confirming parasite species. But there are major causes of blood film negativity in severe malaria, such as recent treatment initiation, inadequate examination of blood films, expertise of microscopist, and identification of non-*falciparum* parasites [4, 19]. Due to this nowadays, platelet parameters including platelet count, MPV, PDW, and PCT, which are the biological markers of thrombocyte morphology and functions, can be obtained or calculated automatically at low cost using hematology analyzers. They are considered to be relatively less important by clinicians for malaria diagnosis [20, 21]. In malaria patients, few studies evaluated platelet index relation with disease severity and prognosis. Then, the current review assessed the role of different platelet indices as a diagnostic marker of severe malaria infection, which can lead to early detection and reduce the severity of malaria infection caused by various species of plasmodium.

## 2. Platelets in Malaria Pathogenesis

The primary function of platelet is regulating hemostasis, but it also plays a secondary role in the innate immune response to infection and the pathogenesis of malaria. This is accomplished by direct killing of the plasmodium parasite and enhancing the sequestration of infected red blood cells (iRBCs) in the vasculature [10]. Platelets also act as classical immune cells by expressing different receptors that bind the host immune response modulators (antibodies and cytokines) and Toll-like receptors that bind the microbial products. Platelets can bind specifically to PF iRBCs, through the interaction between scavenger receptor protein expressed by platelet, CD36, and the PF erythrocyte membrane protein (PFEMP1) which is produced by the parasite and acts as trafficked to the erythrocyte surface. Finally, the iRBCs that bind with platelet are associated with parasite death. Platelets have become active during the early stage of infection in order to slow the exponential growth of malaria parasites in the bloodstream through providing greater chance for activating other body defense mechanisms to control the infection and ensure survival [22].

**Figure 1:**
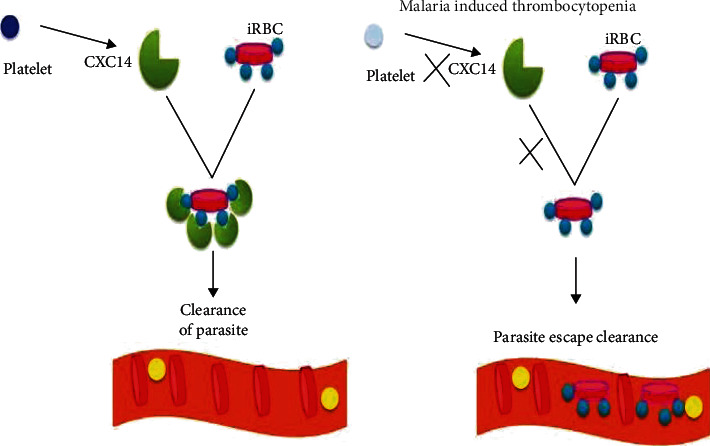
Malaria induced thrombocytopenia (source: Kalyan Srivastava, Monal Sharma, William B Mitchell. Malaria and Thrombopoiesis: A Possible Mechanism for the Malarial Thrombocytopenia, 2017, 2[3]).

As malaria is a hematological disease, all its clinical complications are probably due to its erythrocyte life stage, and consequently, it may interact with the platelets. It was known that platelets are involved in all stages but exceptionally at the hepatic stage of malaria infection. Preferentially, platelets were bound to be infected more than uninfected erythrocytes in the bloodstream and then killing of intraerythrocytic parasites of each plasmodium species specifically mature stages of PV. The spread of parasite pigments is the typical characteristic of dying of platelet-bounded iRBCs [23–25]. The killing of PF parasites through the platelet is necessarily mediated by the platelet factor 4 (PF4 or CXCL4) and erythrocyte Duffy-antigen receptors [26]. Platelet factor 4 is a protein released by activated platelets on contact with parasitized red cells and responsible for the direct killing of intraerythrocytic parasites (Figure 1). Hence, the killing function for PF4 is critically depending on Fy antigen receptors in the red blood cells. However, the genetic disruption of Fy expression inhibits the binding of PF4 to parasitized cells and concomitantly prevents the killing of parasites by both platelets and PF4. Duffy-negative malaria-infected individuals are resistant to platelet-mediated parasite killing, and consequently, the protective function afforded by platelets during malaria infection may be compromised [27, 28].

## 3. Diagnostic Significance of Platelet Indices for Malaria Infection

### 3.1. Thrombocytopenia

Thrombocytopenia, a quantitative alteration in platelets, causes great morbidity. The possible mechanism could explain either peripheral destruction and/or hypoproliferative thrombocytopenia. However, malaria thrombocytopenia is usually due to peripheral destruction of platelets [29, 30]. Most of the time, the high frequency of thrombocytopenia in both PV- and PF-infected patients was almost similar [31]; however, a systematic review and meta-analysis study conducted by Bilal et al. and many more study reports that severe thrombocytopenia (<50 × 10^3^/*μ*L) was the most common manifestation of “severe” PV than PF infection and uncomplicated malaria [15, 32–34]. On the other hand, as severe form thrombocytopenia is a common and early sign of malarial infection, it was more common in *falciparum* malaria, whereas the mild and moderate degree of thrombocytopenia was in PV malaria [35]. A study has been done to support this finding that severe platelet reduction (<50 × 10^3^/*μ*L) was more characterized by *falciparum* (40%) as compared to *vivax* case (11.1%). This reflection indicates that there was a significant association between species of malaria and degree of thrombocytopenia [36]. Thus, the difference frequency of thrombocytopenia between PF and PV infections arose from the different mechanisms by which the pathophysiology of thrombocytopenia is mediated, i.e., clumping in PF and medullar suppression in PV [37]. A better understanding of these mechanisms is still needed, not only by parasite species but also the magnitude of thrombocytopenia depending on the disease severity and degree of parasitemia. The degree of thrombocytopenia is directly proportional to the increase in parasitemia. This may suggest that the extra multiplication of malaria parasites in the host results into platelet destruction. So, this all indicated that patients having thrombocytopenia could be used to determine the presence and severity of malaria infection. Then, it should alert treating physician about the severity of malaria infection [31, 38].

Severe malaria infection may increase thrombocytopenia by 12–15 times compared to uncomplicated malaria. Thrombocytopenia was detected in all severe *falciparum* malaria-infected patients whereas it was seen in about 85% of the patients with uncomplicated malaria. Many studies have been done to support this finding that 80% of patients of either PV or PF malaria develop thrombocytopenia during their infection. In such study, thrombocytopenia has a sensitivity of 83% and a specificity of 68% on malaria-infected individuals [39, 40]. A retrospective study in India showed that thrombocytopenia was the most prominent feature among malaria-infected patients followed by anemia. Thrombocytopenia as a test for malaria diagnosis has the highest sensitivity of 82.43%, specificity of 89.55%, positive likelihood ratio of 7.89, negative likelihood ratio of 0.20, positive predictive value (PPV) of 89%, and negative predictive value (NPV) of 90% [41].

## 4. Platelet Indices

Platelet indices are biomarkers of platelet activation and could be useful for the diagnosis of malaria. They allow extensive clinical investigations focusing on the diagnostic and prognostic values in a variety of settings without bringing extra costs. Platelet indices including PCT, MPV, P-LCR, and PDW are a group of platelet parameters determined by automatic complete blood count profiles, and they are related to platelet morphology and proliferation kinetics [42]. Alteration of these platelet indices will provide us a more comprehensive insight and probable indicators into potential ethology instead of platelet count alone. Various infections and metabolic disorders cause variations in the platelet counts and platelet indices. It was valuable indicators of illness severity, including malaria infection and effective predictors of clinical outcomes. The size of the platelet is decreased as the platelet becomes aged, and an increased MPV indicates an increased proportion of young platelets in the circulation [18, 43].

The normal ranges of MPV, PDW, PCT, and P-LCR for analyzer were as follows: platelet count: 150.00‐450.00 × 103/*μ*L; MPV: 8-12.4 fL; PDW: 9–14 fL; PCT: 0.22-0.24%; P-LCR: 15-35%, respectively [42–44].

### 4.1. Mean Platelet Volume (MPV)

Mean platelet volume is the measure of platelet volume and the most extensively studied markers of platelet activation. It is an indirect measurement of platelet function and activation. Larger platelet size correlates with enzymatically active platelets. Reduced fragmentation of megakaryocytes in the bone marrow and splenic release of larger platelets (increased demand) may increase MPV. However, a decrease in platelet release from the marrow reduces MPV. Platelets change their shape from discoid to spherical and form pseudopodia. This pronounced pseudopod formation leads to increase MPV during platelet activation [45]. Platelet indices were analyzed and increased MPV level for malaria infection. Thus, MPV > 8 fL had a sensitivity and specificity of 70.8% and 50.4% for the diagnosis of malaria, respectively. Due to that, MPV ≥ 9.05 fL was the main predictor for malaria [19, 46]. This is also supported by other studies that MPV > 8 fL showed significant sensitivity of 61.5% with low specificity of 41%. The study also observed that higher MPV (>8 fL) was a more sensitive indicator for PV infection than other plasmodium species, which is similar to a study conducted by Gurudutt and Deepak [16, 34, 47]. There was a strong inverse correlation between platelet counts and MPV. When platelets have been excessively consumed, the bone marrow will produce large amounts of immature platelets, which have a larger volume than mature ones in patients with thrombocytopenia. This may reflect an early release of platelets from the bone marrow in a compensatory response to reduced platelet levels in the peripheral blood [43, 48]. Genetic and acquired factors, such as race, age, smoking status, alcohol consumption, and physical activity can also modify platelet count and MPV [49].

Determination of MPV was found to be extremely useful in ascertaining the etiology of hyperdestructive thrombocytopenia especially malaria. According to this study, a cut-off MPV value of 8.5 fL showed the maximum sensitivity (92.4%) and specificity (100%). The positive predictive value, negative predictive value, and diagnostic accuracy were 100%, 77.78%, and 94%, respectively. Therefore, findings from this study showed that the MPV value of ≥8.5 fL can be used for diagnosing thrombocytopenia cases due to hyperdestructive etiology [50]. This is supported by Pritam et al.'s study; mean MPV was significantly higher in the hyperdestructive group as compared to the hypoproductive group and the control group. They also found that a cut-off MPV value of 7.9 fL would have a sensitivity of 82.3% and a specificity of 92.5% [51]. Accurate measurements of platelet count and size are important for diagnostic, therapeutic, and research purposes. Even though several diseases are associated with MPV abnormalities, some preanalytical variables are affecting the results. Such as the method of venipuncture, choice of anticoagulant, time interval of measurement, temperature, and gently mixing in the sampling tubes may cause platelet activation and produce clumping [52].

### 4.2. Plateletcrit (PCT)

Plateletcrit, which is similar to hematocrit for erythrocytes, is the volume occupied by platelets in the blood as a percentage and calculated according to the formula (PCT = platelet count × MPV/10,000). The normal range for PCT is 0.22–0.24% [42, 46]. It is a measure of platelet mass, which should be interpreted in terms of the number and size of the platelets. The reduction in circulating platelet mass is best explained by a reduction in platelet number rather than a reduction in platelet size. It is suspected that in malaria, sequestration leads to pseudothrombocytopenia. The PCT, MPV, and PDW were significantly lower in the PV cases compared with the PF cases. On the other hand, the PCT was higher in mixed infections compared with PV infections, and the PDW was lower in mixed infections compared with PF infections [37]. Plateletcrit is considered as markers of platelet activation and altered in different clinical conditions including malaria infection. This study also suggests that PCT has a negative correlation with the level of parasitemia; meanwhile, Gupta et al. said a positive correlation was found between platelet count and plateletcrit. The result for plateletcrit with respect to disease severity was indicated by the median plateletcrit, which is 0.023% for a complicated case as compared to 0.06% in an uncomplicated cohort. A plateletcrit of 0.05% had a sensitivity of 65.6% and a specificity of 70.6% to discriminate severe malaria [53, 54]. For the first diagnosis of malaria infection, both platelet count and PCT were the best risk markers with 99% sensitivity and 95% specificity, respectively (Table 1). Therefore, measuring platelet count and PCT is the effective marker for malaria infection and may demonstrate the clinical severity (%malaria) in patients with malaria infection. This study also demonstrated that the increasing level of parasitemia is correlated with the decreasing of both PCT and platelet count [17].

### 4.3. Platelet Distribution Width (PDW)

Platelet distribution width is an indicator of variability of volume in the platelet size and is increased in the presence of platelet anisocytosis. It is used to directly measure variability in platelet size, changes with platelet activation, and reflects the heterogeneity of platelet morphology. Under a normal physiological condition, there is a direct relationship between PDW and MPV. Meanwhile, the relationship between platelet volume and platelet count is not concluded, which suggests that they are affected by different mechanisms [42]. High PDW was observed in malaria-positive than in malaria-negative individuals. Those high values were equally expected in malaria since the excess destruction of platelets would trigger compensatory production of larger platelets, leading to the presence of platelets of varying sizes in the peripheral circulation [56]. On the other hand, raised PDW is predominant indicators of patients with severe PV malaria through longer symptom duration, and the presence of clinical signs and laboratory indicators of severity [57]. Interestingly, Elrazi et al. reported that PDW is the most important hematological predictor of *PF* and *PV* malaria infection. The higher PDW value in malaria could be due to by bone marrow formation of megakaryocytes to compensate for the low absolute platelet count during acute malaria infection. In malaria patients, the production of a significantly higher level of the key platelet growth factor such as thrombopoietin has been reported. Furthermore, excess secretion of dense granules and platelet sensitivity to ADP is increased by the parasitized RBCs [55, 56]. Larger platelets are metabolically and enzymatically more active. Hence, the main mechanism that induces the elevation of PDW during malaria infection is platelet activation [58]. Then, raised PDW in the range of 6-10 showed significant sensitivity of 71.9% with low specificity of 33% for severe malarial infection. The study also observed that PDW 6-10 was more sensitive for PF than PV infection [34]. This is agreed by other researchers that PDW in the range of 6-10 was a more sensitive indicator for *falciparum* (82.6%) than *vivax* (69.5%) malaria [47].

### 4.4. Platelet Large Cell Ratio (P-LCR)

Platelet large cell ratio, the proportion of platelets that have MPV above the upper limit of normal (12 fL), is indicative of the presence of megathrombocytes in circulation [59]. It is the ratio of a number of platelets between 12 fL and the upper discriminator to total platelet count. This ratio provides the variation of which may be used to identify the presence of large platelets. Meanwhile, P-LCR varies inversely with platelet count, and it correlates directly with PDW and MPV [60]. It is a good indicator in the differential diagnosis of conditions associated with abnormal platelet counts if properly utilized. However, P-LCR was found to be significantly higher in hyperdestructive thrombocytopenia patients compared with hypoproductive thrombocytopenia patients, and a cut-off value greater than 33.6% yielded 100% diagnostic sensitivity for hyperdestructive thrombocytopenia. Therefore, it was effective in distinguishing hyperdestructive thrombocytopenia from hypoproductive thrombocytopenia [26]. A significantly higher P-LCR value was reported among malaria-positive than malaria-negative patients. This is also an expected finding in malaria as the proportion of large platelets in circulation commonly increases following peripheral destruction leading to a higher P-LCR [56].

### 4.5. Immature Platelet Fraction (IPF)

Immature platelet fraction indicates the percentage of immature platelets, as a percentage of the total platelet population measured in the reticulocyte platelet channel of the hematology analyzer by a flow cytometer. The IPF percentage increases as the production of platelets increases, and low values indicate suppressed thrombopoiesis [61]. Reticulated platelets are immature platelets which are newly released from the bone marrow and, like their red cell counterpart (reticulocytes), are rich in RNA and larger in size. The IPF% is raised in conditions with increased peripheral destruction such as immune thrombocytopenia in malaria infection. An IPF% of 7.7% is the best point for the highest sensitivity (86.8%) and specificity (92.6%) in the diagnosis of ITP and the recovery phase postchemotherapy. This also found that the IPF% rose as thrombocytopenia developed in early malaria, and it was significantly more useful than the MPV [62, 63]. In human malaria infection, the decrease in platelet numbers was associated with a concurrent rise in young platelets (immature platelet fraction) and thrombopoietin. Platelet production was assessed by measurement of IPF, a parameter for young platelets that was recently introduced on Sysmex analyzers [24]. Renuka et al. had also observed a positive correlation between the IPF level and the recovery of platelets in patients with malaria [64].

## 5. Conclusion

Although platelet indices are easy to perform, inexpensive, and involved in routine hematological examinations, they are considered to be relatively less important by clinicians for predicting malaria severity. The present study is menacing that determination of platelet count and platelet indices had significant sensitivity and specificity to identifying malaria severity. Elevation of MPV and PDW with decreased PCT and platelet counts is a useful predictor of malaria severity. The IPF% is raised in conditions with increased peripheral destruction such as in the case of severe malaria, and it was significantly more useful than the MPV. Further studies are needed to explore and validate the utility of platelet indices as a marker of malaria severity prior to the use of these indices.

## Figures and Tables

**Table 1:** Platelet count and platelet indices for diagnosis in patients with malaria infection.

Author and year	Study area	Study design	Sample size		Parameter			
					P.count (/*μ*L)	PCT (%)	MPV (fL)	PDW (%)
Tangvarasittichai et al., 2016 [17]	Thailand	Cross-sectional	100	Cut-off value	50 × 10^3^	0.119	8.55	15.75
				Sen%	97	94	78	79
				Spe%	96	99	79	80
Chandra and Chandra, 2013 [34]	India	Retrospective	334	Cut-off value	150 × 10^3^		>8	>10
				Sen%	87.2		61.5	71.9
				Spe%	65		41	43
Salih et al., 2018 [55]	Sudan	Case control	Case 67 & 105 control	Cut-off value	200 × 10^3^			15.34
				Sen%	80.7			80.1
				Spe%	75.0			66.3

NB: Sen = sensitivity; Spe = specificity; P.count = platelet count.

## Data Availability

All relevant data are fully available without restriction within the manuscript.

## Abbreviations

Fy: Duffy-antigen receptor
iRBCs: Infected red blood cells
MPV: Mean platelet volume
NPV: Negative predictive value
PCT: Plateletcrit
PDW: Platelet distribution width
PF: *Plasmodium falciparum*
PF4: Platelet factor 4
P-LCR: Platelet large cell ratio
PPV: Positive predictive value
PV: *Plasmodium vivax*
RBCs: Red blood cells.
